# Safety and efficacy of using portable coagulation monitor for INR examination after left-sided mechanical prosthetic valve replacement

**DOI:** 10.1186/s13019-022-02046-8

**Published:** 2022-12-05

**Authors:** Yue Shen, Fu-xiu Zhong, Xue-shan Huang

**Affiliations:** 1 Department of Cardiovascular Surgery, Union Hospital, Fujian Medical University, Fuzhou, 350001 People’s Republic of China; 2 Fujian Key Laboratory of Cardio-thoracic Surgery, Fujian Medical University, Fuzhou, People’s Republic of China; 3 Department of Surgery, Union Hospital, Fujian Medical University, Fuzhou, 350001 People’s Republic of China

**Keywords:** Coagulation monitor, Warfarin therapy, Mechanical prosthetic valve

## Abstract

**Background:**

Time in therapeutic range (TTR) is an index to assess the effectiveness of anticoagulation and is important to predict the risk of bleeding and thrombosis in patients taking warfarin. In recent years, the portable coagulation monitor, a point-of-care testing device for patients to perform self-management international normalized ratio (INR) examination, has provided an opportunity to improve the quality of oral warfarin treatment. In this study, we applied TTR to evaluate the safety and efficacy of the portable coagulation monitor for patients with oral anticoagulant warfarin after left-sided mechanical prosthetic valve (MPV) replacement.

**Methods:**

It is a single-centre cohort study. From September 2019 to June 2021, a total of 243 patients who returned to our institution for outpatient clinic revisit at 3 months after left-sided MPV replacement, met the inclusion criteria and agreed to be followed up were included. Self-management group used portable coagulation monitor for INR examination, and patients in the conventional group had their INR monitored in routine outpatient visits. Clinical data of the patients would be recorded for the next 12 months, and results were compared between the two groups to assess the effect of the coagulation monitor on TTR and complications related to bleeding and thrombosis in patients with left-sided MPV replacement.

**Results:**

A total of 212 individuals provided complete and validated INR data spanning of 1 year. Those who applied the portable coagulation monitor had higher TTR values and larger number of tests for INR. No significant differences were seen between the two groups in postoperative bleeding and thromboembolic complications, but portable coagulation monitor showed a trend toward fewer bleeding events.

**Conclusion:**

Portable devices for coagulation monitoring are safe and can achieve a higher TTR. Patients who use the portable coagulation monitor for home INR self-management can achieve a safe and effective warfarin therapy.

## Introduction

Valvular heart disease is the most common type of cardiovascular disease and its prevalence is increasing dramatically with the ageing of the population. In recent years, the number of prosthetic valve replacement has also shown a year-on-year increase [1]. Following mechanical valve replacement, patients are required to receive anticoagulation therapy with oral warfarin for life to prevent thrombosis. Due to the narrow target therapeutic range of anticoagulation, regular monitoring of the INR is required during anticoagulation with warfarin to maintain the target level of anticoagulation, and to avoid serious side effects such as bleeding due to overdose [2].

The efficacy of warfarin is influenced by a number of factors. Its slow onset of action, numerous dietary, herbal and drug–drug interactions tend to cause large fluctuations in anticoagulation levels. Large fluctuations in anticoagulation levels that deviate from the normal target therapeutic range are likely to have adverse consequences and are detrimental to the patient’s prognosis. Previous studies have demonstrated that patients with unstable anticoagulation levels have a less favourable prognosis than those with stable anticoagulation levels [3, 4].

High quality anticoagulation management is therefore needed, to make warfarin, a drug with a narrow therapeutic range, as effective and safe as possible, and clinical indicators are also needed to be introduced to assess the effectiveness of anticoagulation in patients and to reduce the risk of poor anticoagulation control. In recent years, TTR has been introduced to assess the effectiveness of anticoagulation with warfarin in patients. TTR is the percentage of time that a patient’s INR is within the desired therapeutic range and is widely used as an indicator to assess the stability of anticoagulation control with warfarin [5].

A multicentre study showed that TTR was associated with anticoagulation-related mortality, thrombosis and bleeding after mechanical valve replacement, with a TTR of less than 70% corresponding to double the rate of bleeding and mortality and a 50% higher rate of thromboembolic events compared with a TTR of greater than 70% [6]. Therefore, during anticoagulation treatment with taking warfarin, it is important to pay attention not only to the INR but also to the anticoagulation effectiveness indicator TTR, which is defined as effective for ≥ 70% according to the current European guidelines, and TTR < 70% is considered to be of low quality [7, 8].

Because the clinical efficacy of warfarin is affected by a number of factors, coagulation monitoring and dose adjustment are required for taking warfarin. Patients require frequent clinic visits for INR testing, causing serious inconvenience, reducing patient compliance [9], increasing the risk of bleeding [10, 11], and leading to a significant proportion of patients not achieving adequate level of anticoagulation. In recent years, therefore, the portable coagulation monitor has been popularized worldwide as a device designed to enhance patient compliance with self-management, with the advantages of portability and ease of use. The results can be taken in a few minutes, eliminating the need for patients to travel from home to hospital, reducing the waiting time for the examination and enhancing patient compliance.

The aim of our study was to compare the TTR of the two groups of patients, as well as anticoagulation-related complications, and thus to assess the effectiveness (TTR) and safety (bleeding, embolism and other complications) of applying a portable coagulation monitor to guide anticoagulation therapy with warfarin.

## Materials and methods

### Patients

Between September 2019 and June 2021, there were 243 consecutive patients who have undergone aortic and/or mitral valve replacement surgery and re-visited to our outpatient clinic at 3 months after surgery were enrolled. All enrolled patients were required to receive anticoagulation therapy with oral warfarin for life to prevent thrombosis after mechanical valve replacement. Patients who underwent left-sided mechanical prosthetic valve replacement during this period were reviewed for their electronic medical records. The following criteria needed to be met by the included participants: (1) aortic and mitral mechanical valve replacement; (2) stable physical condition; and (3) received oral warfarin therapy. The exclusion criteria included: (1) patients requiring additional antithrombotic medication, such as those who have undergone cardiovascular surgery, comorbid lower limb venous thrombosis and pulmonary embolism, and various other conditions requiring antiplatelet agents and heparin, (2) severe disease of digestive and the nervous system, (3) Based on previous literature, we excluded patients with greater than 8 weeks between determination [12], (4) Patients who refuse to be followed up.

### TTR

TTR was assessed using the Rosendaal method, which is based on linear interpolation. This method assumes that there is a linear relationship between successive observed INR values, with each individual being assigned a specific INR value daily. TTR is calculated as the percentage of the assigned INR value that remains within the treatment range [13]. In clinic practice, the most commonly used method for calculating individual TTR is percent of visits in range, which is the percentage of total follow-up visits that achieve the target INR (usually the INR for the first 6 weeks of the initial warfarin application is not calculated), e.g. if you have 7 out of 10 outpatient follow-up visits in 6 months and your INR is within the therapeutic range, your TTR is 70%.

### Measurement methods

The administration of warfarin requires frequent INR monitoring to ensure anticoagulation is within the therapeutic range. However, the therapeutic range of warfarin is narrow and has a large inter-patient variability, and there is no identical optimal level of anticoagulation (target INR) for different patient groups [14, 15]. In China, patients usually receive low-intensity oral warfarin anticoagulation (target INR < 2–3). For the Chinese patients, target INR was considered to be relatively low because they are more sensitive to warfarin treatment and have higher risk of bleeding events than whites [16, 17]. In the Chinese population, low-intensity warfarin anticoagulation was proven to be effective in preventing thromboembolic events and was associated with fewer major bleeding complications [18–20]. Our patients were mainly from southern China and the target treatment range in this study was 1.5–2.0 for aortic valve replacement, 1.7–2.3 for mitral valve replacement and for aortic and mitral valve replacement. If the patient’s INR was greater than 3 then vitamin K1 was administered or warfarin was temporarily stopped and the INR was tested again the next day.

Patients routinely re-visited our institution for blood tests, coagulation function, ECG, chest radiograph and echocardiogram at 3 months after surgery. Patients who met the inclusion criteria and agreed to be followed up had their INR and other indicators collected over a period of 1 year. All the patients were tested for INR every 2 weeks to 1 month. Patients who choose to be followed up in the outpatient clinic were enrolled into the conventional group, and based on the results of the test, the follow-up department is contacted for anticoagulation instructions. Patients who choose to perform their own INR tests at home using a portable device and receive dose instructions from the follow-up department or manage their own dose were included in the self-management group.

### Complications

Clinical complications and outcome events for this study as defined by Guidelines for reporting mortality and morbidity after cardiac valve interventions and ESCAT study [21–23].

The ESCAT study classifies bleeding and thromboembolic complications into 3 classes. Grade III bleeding and thromboembolic complications that occur during the follow-up period would be recorded. Grade III bleeding is defined as serious bleeding requiring blood transfusion, surgical or endoscopic intervention, hospitalisation or leading to long-term damage. Grade III thromboembolism is defined as serious thromboembolism requiring hospitalisation or leading to long-term damage, resulting in, for example, transient ischaemic attack and thrombosis of a heart valve prosthesis.

### Statistical analysis

IBM SPSS statistics software version 22 (IBM Corp., SPSS Inc.) was used for statistical analysis, and significant differences were defined as *p* values < 0.05. Quantitative data with a normally distribution was reported as the mean ± one standard deviation, median with interquartile range (IQR) were used for non-normal distribution data. The student's t-test was used to compare normally distributed continuous variables, and the Mann–Whitney U-test was used to compare non-normally distributed continuous variables. For categorical data, the chi-square test or Fisher test was used.

## Results

Of the 243 patients, 31 (12.76%) dropped out for various reasons before the study was completed. Five patients (8.47%) dropped out in the self-management group and twenty-six (14.13%) in the conventional group, with a higher dropout rate in the conventional group.

During the follow-up period, two patients were hospitalized for coronary percutaneous coronary artery intervention therapy, one patient received carotid artery stenting and coronary percutaneous coronary artery intervention therapy, one patient with lower extremity arterial stenosis, and one have undergone previous abdominal procedures and all of the appealed patients were administered with additional antithrombotic drugs such as low-molecular-weight heparin or aspirin.

There were 212 patients who provided complete and valid coagulation profile. A total of 54 patients underwent coagulation testing using the coagulation monitor, and 158 patients were tested in the hospital. There were no differences in patient baseline data, and no significant differences were seen in the type of surgery between the two groups. A total of 44 patients were replaced with CarboMedics mechanical prostheses, 76 patients with ATS mechanical prostheses, 100 patients with St. Jude Medical mechanical prostheses, and 21 patients with St. Jude regent mechanical prostheses. No differences were found between the two groups for concomitant Cox-Maze surgery and concurrent tricuspid annuloplasty. There were still 72 patients with combined atrial fibrillation on the outpatient review at the 3rd month after surgery, and no difference was found between the two groups. No significant differences were found in the indices of cardiac function and other indicators on review. All baseline and follow-up data are shown in Table 1.

**Table 1:** Baseline data and follow-up data at 3rd -month postoperative

Item	Self-management	Conventional	*p*
Female, n	26	81	0.692
Age (years), mean ± SD	52.01 ± 11.84	51.49 ± 11.19	0.832
BMI (kg/m²), mean ± SD	22.35 ± 2.01	22.51 ± 2.33	0.874
Diabetes, n	5	17	0.755
Hypertension, n	10	41	0.270
Surgical type, n			0.895
Mitral valve	27	76	
Aortic valve	19	61	
Mitral and aortic valve	8	21	
Prosthetic valve type, n			0.521
CarboMedics	15	29	
ATS	18	58	
St-Jude medical	23	77	
St-Jude regent	6	15	
Cox-Maze procedure, n	9	22	0.622
Tricuspid annuloplasty, n	14	32	0.142
Endoscopic mitral surgery, n	12	31	0.331
Atrial fibrillation, n	17	55	0.656
LVED (%), mean ± SD	59.45 ± 6.47	58.92 ± 6.13	0.638
LVEF (%), mean ± SD	62.14 ± 7.99	61.98 ± 7.76	0.722
NYHA, median (IQR)	1 (0)	1 (0)	0.784

*BMI* body mass index, *LVEF* left ventricular ejection fraction, *LVED* left ventricular end diastolic diameter, *NYHA* New York Heart Association classification

Table 2 showed that the number of coagulation tests was significantly higher in the self-managed group compared with the conventional outpatient clinic visit group. There were no valve disease-related deaths during follow-up, and a total of 11 patient/year grade III complications (bleeding and thromboembolic events) occurred throughout the follow-up period. There were 8 bleeding events and 3 thromboembolic events, accounting for 5.66% of the annual incidence of all patients studied. Gastric bleeding occurred in one patient in the self-management group while taking warfarin, compared with 7 in the conventional group (2 cerebral hemorrhages, 4 gastrointestinal hemorrhages, and 1 subdural hematoma); 1 embolic event occurred in the self-management group and 2 in the conventional group (1 cerebral infarction and 1 lower extremity deep vein thrombosis).

**Table 2:** Follow-up data

Item	Self-management	Conventional	*p*
INR test numbers, median (IQR)	23 (8.25)	22 (8)	0.039
Medical therapy			
Amiodarone	16	44	0.802
Digoxin	35	99	0.777
β-blocker	45	9	0.391
Operated valve endocarditis, n	0	0	NS
Structural valve deterioration, n	0	0	NS
Valve thrombosis, n	0	0	NS
Embolism event, n	1	7	0.683
Bleeding event, n	1	2	1
TTR (%), mean ± SD	0.76 ± 0.13	0.72 ± 0.12	0.19

## Discussion

Warfarin is the most widely used oral anticoagulant in the world. Patients with prosthetic mechanical valve implantation require lifelong oral warfarin for anticoagulation therapy [2]. Warfarin is very effective when anticoagulation levels are maintained within the target therapeutic range, but exceeding the target therapeutic range may cause serious consequences, such as anticoagulation-related bleeding and thromboembolism, which can even lead to death in severe cases [7].

However, because of a variety of factors, the INR value often fluctuates during warfarin administration, requiring regular coagulation monitoring and dose adjustment in clinical practice. Patients are subject to require frequent visits to the clinic for coagulation testing. Some patients often reduce the frequency of testing or even do not monitor INR at all due to the great inconvenience it causes to their lives, which in turn reduces compliance with warfarin anticoagulation control, resulting in a failure to achieve anticoagulation or even a serious bleeding event [9, 24].

Due to the limitations of warfarin anticoagulation, in order to assess the effectiveness of taking warfarin anticoagulation, it is important to focus not only on INR but also on the overall quality of anticoagulation while taking warfarin. TTR is widely used as an indicator to assess the quality of anticoagulation therapy and is the gold standard used to assess the effectiveness of warfarin anticoagulation as the percentage of time a patient’s INR is within the desired therapeutic range [25]. Previous Studies have indicated that the real benefit of warfarin treatment is achieved by controlling TTR above 70%, a level that provides protection against thromboembolism without increasing the risk of bleeding [7, 8]. Our patients in both groups had a TTR close to 70%. Patients using the portable device had a higher TTR than those who revisit the clinic for coagulation testing, with a significant difference between the two groups. This indicates that patients using the portable device had better results with anticoagulation therapy.

We found that the total number of measurements was greater in patients with portable devices in this study. The availability of portable devices and home monitoring has facilitated more frequent testing. Clinical evidence suggests that more frequent testing will lead to tighter anticoagulation control, which may improve the safety of warfarin and reduce the risk of thromboembolic and major bleeding events [26].

A clinical practice guideline states that patient self-management may be recommended over routine outpatient INR monitoring for patients treated with warfarin who are motivated and can demonstrate their ability to self-manage (including self-testing devices) [27]. Surveys have shown that patient compliance is an important factor in TTR. If patient compliance is high, then TTR is usually higher [28]. The use of coagulation monitors for patient self-management enhances patient compliance as it does not require frequent trips to the hospital and the monitoring results are simple, quick and easy to obtain, which may be associated with their higher TTR. The study also showed that patients who implemented home monitoring tended to have INRs in the therapeutic range and lower rates of complications and hospitalizations than patients who had their INR monitored in the clinic visit. Patients who apply portable devices for self-management have a slightly reduced risk of death, thromboembolic risk, and risk of major bleeding. The main barrier to widespread use of patient self-management is cost, with test equipment plus a set of test strips costing thousands of dollars. This also suggests that patients who are willing to spend on equipment tend to be in a better financial position and more willing to spend on health [2]. The coagulation monitor is therefore an important aid for patients taking warfarin for the prevention of thromboembolism, where the patient’s financial situation permits. The coagulation monitor is easy to use, quick to obtain results, improves patient compliance and facilitates the adjustment of the patient’s warfarin dose, thus ensuring that the patient can truly benefit from taking warfarin.

Previous literature has shown that offering medication guidance to patients with mechanical heart valve replacement via telemedicine is safe and feasible [29]. Our institution is located in a province with many mountains and islands proximity to the coastline, a region with a population of 41.54 million according to the census, which is slightly inaccessible due to the barrier of mountains and waters. Our institution is the main centre for cardiac surgery in the region, while the rest of the region has no experience of post-operative patient management and cannot effectively assist patients. The use of the coagulation monitor and the use of smartphone communication software for follow-up visits can greatly reduce the burden of travel on patients and increase their compliance. In addition to improving patient compliance, the coagulation monitor provides immediate results so that doctors can be informed if an anticoagulation overdose is the cause. During the follow-up period of our study, a patient contacted us with unexplained black stools and the coagulation monitor measured an elevated INR. We immediately advised the patient to seek medical intervention for excessive anticoagulation, and reduced the onset to treatment time.

As China is an ageing and urbanising society, there are many elderly patients who live alone in the countryside and we have found in our clinical practice that most of these patients do not have young educated family members to accompany them after surgery. With the coagulation monitor, it is easy to use and saves the hassle of hospital visits for INR testing. Family members can remind the patient by phone or supervise the test via video communication software, enhancing patient compliance.

### Limitation

Despite the results obtained in the study, however, there are still some limitations. Firstly, our study did not limit the type of coagulation testing devices purchased by patients, the small sample size of the study, and the inherent shortcomings of the TTR calculation method do not reflect the variability of the INR.

Second, the higher compliance of those who agreed to the follow-up study caused an overestimation of TTR values, which would be higher than the general population in the natural state, with selection bias.

Nevertheless, our comparisons between populations in the same region were consistent, and baseline data such as diabetes, age, and cardiac function did not differ between the two groups of patients, which still have some clinical significance.

## Conclusion

The use of portable coagulation monitors for anticoagulation in patients could increases patient compliance. Patients using portable devices for INR monitoring and self-management may achieve a safe and more effective warfarin treatment.

## Data Availability

Data sharing was not applicable to this article, as no data sets were generated or analysed during the current study.

## Abbreviations

TTR: Time in therapeutic range
INR: International normalized ratio
MPV: Mechanical prosthetic valve
IQR: Interquartile range
BMI: Body mass index
LVEF: Left ventricular ejection fraction
LVED: Left ventricular end diastolic diameter
NYHA: New York Heart Association classification
