# Correction: Safety and efficacy of using portable coagulation monitor for INR examination after left‑sided mechanical prosthetic valve replacement

**DOI:** 10.1186/s13019-022-02076-2

**Published:** 2022-12-15

**Authors:** Yue Shen, Fu‑xiu Zhong, Xue‑shan Huang

**Affiliations:** 1 Department of Cardiovascular Surgery, Union Hospital, Fujian Medical University, Fuzhou, 350001 People’s Republic of China; 2 Fujian Key Laboratory of Cardio‑thoracic Surgery, Fujian Medical University, Fuzhou, People’s Republic of China; 3 Department of Surgery, Union Hospital, Fujian Medical University, Fuzhou, 350001 People’s Republic of China

Correction to: Shen et al. Journal of Cardiothoracic Surgery (2022) 17:297 10.1186/s13019-022-02046-8

Following the publication of the original article [1], in "Author Contribution" section author name needs to be expanded. So the text will read as follows:

Yue Shen and Fu-xiu Zhong contributed equally to this study and share first authorship. Xue-shan Huang and Yue Shen designed the study, participated in the operation, and drafted the manuscript. Yue Shen and Fu-xiu Zhong collected the clinical data and performed the statistical analysis. Xue-shan Huang provided technical support. All authors read and approved the final manuscript.

The original article has been corrected.
